# Bilateral Blindness Following Chemoradiation for Locally Advanced Oropharyngeal Carcinoma

**DOI:** 10.7759/cureus.352

**Published:** 2015-10-15

**Authors:** K. Liang Zeng, Sara Kuruvilla, Michael Sanatani, Alexander V Louie

**Affiliations:** 1 Faculty of Medicine, University of Toronto, Toronto, Canada; 2 Medical Oncology, Schulich School of Medicine & Dentistry, London Regional Cancer Program, Western University, London, Ontario, CA; 3 Department of Radiation Oncology, London Regional Cancer Program, Western University, London, Ontario, CA

**Keywords:** wernicke's encephalopathy, thiamine, blindness

## Abstract

Wernicke's encephalopathy is a life-threatening neurologic complication of thiamine deficiency. Though the presentation of symptoms can vary widely, the classical triad is founded on ophthalmoplegia, alteration of mental status, and gait disturbance. We describe a case of Wernicke's encephalopathy in an oncology patient shortly after concurrent 5-fluorouracil, carboplatin, and radiotherapy for locally advanced oropharyngeal cancer, presenting as complete bilateral blindness, ataxia, nystagmus, and confusion. Thiamine was given based on clinical suspicion and rapid improvement of clinical findings occurred. An MRI performed later supported the diagnosis of Wernicke's encephalopathy. A multifactorial etiology of thiamine deficiency from nutritional deficits and neurotoxic effects of chemotherapy are hypothesized.

## Introduction

Central neurotoxic side-effects are a rare consequence of chemotherapy; however, they are more prevalent in patients treated with methotrexate, cytarabine, and ifosfamide [1]. These toxicities can range from minor cognitive deficits to encephalopathy, to progressive dementia, and even coma.

Wernicke’s encephalopathy is a well-described neurologic complication of thiamine (vitamin B1) deficiency. Although most commonly seen in the setting of chronic alcoholism, alternate mechanisms can include poor nutrition, poor absorption, reduced dietary intake, or increased metabolic requirement, as is the case in certain systemic illnesses [2]. Wernicke’s encephalopathy is characterized as an acute syndrome of ataxia, ophthalmoplegia, and confusion, and emergent treatment is warranted to prevent long-term neurologic consequences and even death.

5-Flurouracil (5-FU) inhibits DNA synthesis via inhibition of thymidylate synthase by the metabolite 5-fluoro-2-deoxyuridine-5-phosphate. It is commonly used in the treatment of malignancies arising from the breast, rectum, and colon. Common sequelae from treatment with 5-FU include suppression of bone marrow resulting in leukopenia and symptoms due to disruption of the gastrointestinal epithelium (i.e., nausea, vomiting, diarrhea, and anorexia). Neurotoxic side-effects and, in particular, central neurotoxic side-effects are rare [3].

Carboplatin is a platinum-based chemotherapeutic that acts by crosslinking DNA and thereby inhibiting DNA repair and/or synthesis. Commonly used in head and neck cancers, it can also lead to myelosuppression and gastrointestinal side-effects. Central neurotoxicity is exceedingly rare, although long-term peripheral neuropathies have been reported [4].

## Case presentation

A recently treated 55-year-old oncology patient was admitted to our hospital for severe malnutrition and 44 kg weight loss (36% of body weight) over six weeks.

Forty days prior, he had completed a course of concurrent radiotherapy (70 Gy in 35 fractions) and 5-fluorouracil and carboplatin for squamous cell carcinoma of the tonsil. High-dose cisplatin is recognized as the standard systemic treatment regimen administered concurrently with radiation for this indication; however, this alternate regimen as per Calais, et al. was selected due to significant patient aversion to potential hearing deficits [5]. Prior to the start of chemotherapy and radiotherapy, full-mouth tooth extraction took place due to poor dentition. 

The first cycle of concurrent chemoradiotherapy was tolerated well with no adverse events. Towards the end of his therapy, he struggled with Grade 2 (NCI CTCAE v4) odynophagia, requiring supplementation with a fluid diet and dietary consultation. He had documented weight loss from 122 kg to 101 kg while on treatment. He continued to receive dietary support, fluid supports, and non-oral analgesia upon completion of his treatment.

In the period between completion of concurrent chemoradiotherapy and presentation to hospital, he was on disability leave and recovering at home. In retrospect, he stated that he continued having difficulty with swallowing and nausea/vomiting. He resisted the idea of having a feeding tube inserted and did not seek medical attention until urged to by family and health-care workers.

As part of his initial assessment, it was difficult to obtain a clear timeline of events and it became apparent that his clinical condition extended beyond malnutrition. He began to develop gradual visual deficits over three to four days that progressed to complete bilateral blindness.

Physical examination revealed normal vitals, disorientation, confusion, and truncal ataxia. He was unable to perceive light or motion and did not blink to threat. Extraocular eye movements were restricted to both right and left gaze, and there was evidence of changing nystagmus with all directions of gaze. Funduscopic examination revealed normal posterior segments bilaterally. Neurological examination was otherwise unremarkable. His presenting weight was 78 kg (a 36% weight loss from initial presentation prior to treatment). There was no family history of vasculitides, congenital visual disorders, or neurologic disorders. He drank alcohol occasionally.

Initial laboratory investigations demonstrated normal complete blood count and differential. Albumin was 32 g/L (normal: 35-50 g/L). Liver enzymes and renal function tests were normal. Initial folate and vitamin B12 were also normal. Toxicology did not identify any illicit substance use.

**Figure 1:**
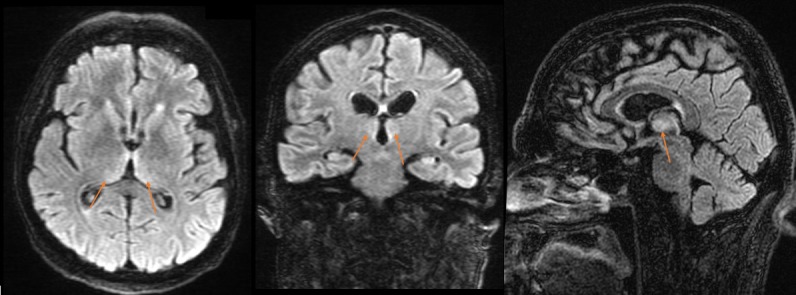
MRI head: axial, coronal, and sagittal FLAIR sequences, demonstrating periventricular hyperintensities at the medial thalami bilaterally, consistent with Wernicke’s encephalopathy. Orange arrows identifing areas of abnormality.

Based on the clinical findings and history, a diagnosis of Wernicke’s encephalopathy was made and thiamine was given based on clinical suspicion. He was treated with 100 mg thiamine IV daily for five days and titrated to a maximum dose of 250 mg IV daily. Symptomatic improvement with thiamine was rapid, with visual acuity improvement to 20/200 OU in three days. An MRI performed three days later demonstrated signal abnormalities at the periventricular region of the third ventricle and medial thalami bilaterally (Figure 1), adding further support to the diagnosis.

Cognition and ataxia improved slowly during the hospital stay. After consultation with our dietician service, nutritional supplementation via percutaneous gastrostomy tube was instituted after initial hesitation by the patient. His weight increased to 86.6 kg three weeks after admission. Prior to repatriation to his home hospital, visual acuity improved to 20/70 OU. Evidence of persistent neurologic deficits was still present at the time. 

## Discussion

We present a case of a 55-year-old man treated recently with concurrent chemotherapy and radiotherapy for locally advanced oropharyngeal carcinoma, presenting with bilateral blindness, ataxia, confusion, and nystagmus. Based on the history, clinical findings, and imaging, a diagnosis of Wernicke’s encephalopathy was made. Rapid neurologic improvements were seen with thiamine supplementation. Nutritional intake is challenging for patients receiving combined chemoradiotherapy, and this case demonstrates an extreme case of Wernicke’s encephalopathy as a result of treatment.

Central neurotoxic side-effects from 5-FU are rare, and even rarer from carboplatin. Pirzada, et al. and Yeh and Cheng report an approximately 5% rate of neurotoxicity and encephalopathy from 5-FU [3, 6]. Though Wernicke’s encephalopathy has previously been reported in patients receiving 5-FU, presentation with a profound visual impairment has not previously been reported [7-11].

The pathophysiology of Wernicke’s encephalopathy is based on the role of thiamine and its function as a cofactor for enzymes in the brain. Impaired activity of enzymes dependent on thiamine (especially alpha-ketoglutarate dehydrogenase complex – α-KGDG) can ultimately lead to neuronal necrosis and irreversible structural lesions in the brain, via a combination of changes in the levels of amino acid production, astrocyte edema, and focal acidosis [2]. Thiamine stores are depleted within 18 days in the body. These changes can initially be reversible by restoring thiamine levels, but neurologic deficits may become permanent once permanent lesions have developed.

Patients presenting with Wernicke’s can manifest a wide range of symptoms. It is difficult to diagnose at subclinical levels of thiamine deficiency as nonspecific symptoms such as fatigue, irritability, and headaches may be present. The classical symptoms of Wernicke’s encephalopathy of mental status change, motor issues, and ophthalmoplegia are a result of involvement in the thalamic and/or mammillary bodies [12]. Though primarily a clinical diagnosis, MRI plays a role in supporting this diagnosis. MRI has a specificity of 93% and typically demonstrates symmetrically increased T2 signals in the periventricular regions of the thalamus, periaqueductal region, hypothalamus, mammary bodies, and floor of the fourth ventricle [13].

There are two mechanisms which may have contributed to our patient developing thiamine deficiency and subsequently Wernicke’s encephalopathy: (1) nutritional deficiency and (2) as a result of the 5-FU administration itself. Certainly, the significant weight reduction (36% of original body weight lost) and hypoalbuminemia support poor intake during this time period, suggestive of nutritional thiamine deficiency. Additionally, it has been postulated that 5-FU can interfere with thiamine bioavailability. Encephalopathy alone can result from interference with the Krebs cycle by fluoroacetate, a product of 5-FU catabolism, leading to a high ammonia state [14]. Through a second mechanism, 5-FU has been recognized to decrease the formation of thiamine pyrophosphate, the active form of thiamine [3, 15]. Rapid thiamine supplementation has been shown to reverse thiamine deficiency caused by 5-FU administration alone. It is possible that nutritional thiamine deficiency was compounded by 5-FU neurotoxic and blockage of thiamine-phosphate effects, leading to our patient’s presentation. Delineating between the two contributors is difficult, if not impossible.

The rapid response to thiamine supplementation alone and visual symptom improvement is in keeping with what has been reported previously. Cho, et al. describe a similar case, where a patient receiving concurrent chemotherapy (5-FU) and radiation for head and neck cancer, presented with Wernicke’s encephalopathy in the setting of deficient nutritional intake shortly after treatment [10]. The patient’s mental state improved after several hours, and further improvements in nystagmus and dizziness gradually improved over the next few days.

Though there is clinical and radiographical support of Wernicke’s encephalopathy, biochemical evidence was, unfortunately, unavailable to us. Serum thiamine concentration has previously been measured in patients with presumptive Wernicke’s encephalopathy. However, its use clinically is limited by lack of specificity and technical factors [2]. In one similar case report, the thiamine concentration was actually greater than the upper limit of normal [7]. Newer methods, including high-performance liquid chromatography, may be more consistent in reflecting thiamine levels [16]. These assays are not standard in current clinical practice.

Management of patients receiving combined chemoradiation for head and neck malignancies is complicated by high rates of comorbidities, high risks of psychosocial distress, and sequelae to highly toxic therapies. With improved outcomes, these patients experience complex survivorship issues and often require multidisciplinary teams during treatment and during the years after treatment to address potential co-morbidities [17]. Dedicated teams that recognize such potential issues may play an important role for these patients. 

## Conclusions

Our case demonstrates an extreme example of Wernicke’s encephalopathy that was caused by a severe nutritional deficiency (36% weight loss over two months) accentuated by prior exposure to 5-FU. Initial supplementation was difficult due to the patient refusal of a feeding tube several times early in treatment as well as during his recent admission. Care of patients after curative treatment for head and neck cancers is complicated by the high frequency of comorbid diseases, psychosocial distress, and sequelae of the treatment itself. Long-term specialized care is likely needed for patients after treatment of head and neck cancers for surveillance, follow-up, and management of chronic morbidity from treatment. Multidisciplinary care including a medical oncologist, radiation oncologist and dietician are important in addressing potential nutritional deficiencies during and after treatment to prevent serious complications.
